# Non-canonical and constitutive activation of small GTPases: more than an exception to the rule?

**DOI:** 10.1042/BCJ20250137

**Published:** 2026-06-04

**Authors:** Jasmine Kiers, Peter L. Hordijk

**Affiliations:** Department of Physiology, Amsterdam UMC, Amsterdam Cardiovascular Sciences, Atherosclerosis and Aortic disease, O2 Science building, 10W51, De Boelelaan 1108, 1081HZ Amsterdam, The Netherlands

**Keywords:** lactylation, palmitoylation, Ras GTPases, Rho GTPases, ubiquitination

## Abstract

The superfamily of small GTPases comprises over 150 members, clustered in different families (e.g., Ras, Rab, Rho), that together are essential for a wide range of cellular functions. Most GTPases behave as molecular on/off switches, with regulatory proteins controlling their cycling between the GDP-bound, inactive form and the GTP-bound, active form. However, there is a substantial number of small GTPases that do not adhere to this canonical mode of regulation. This can result from key sequence differences that maintain these GTPases mostly in the GTP-bound form. Alternatively, specific post-translational modifications, including ubiquitination or alternative lipidation, can activate these GTPases, for example, due to interference with their intrinsic GTP hydrolysis capacity. Such ‘atypical’ GTPases are often constitutively active and are furthermore characterized by fast turnover. Consequently, their output is much more a function of their expression levels, with proteasomal or lysosomal degradation limiting their signaling capacity. This review discusses recent insights into non-canonical activation of small GTPases. We also discuss modifications, in particular palmitoylation, which allows otherwise canonical GTPases to become (constitutively) active, escaping their usual regulatory mechanisms. Finally, we discuss the potential biological importance of non-canonical GTPase regulation. We focus on the difference in kinetics and signal duration between canonical cycling versus non-canonical activation, and discuss differences related to fast, external stimulation (e.g., by hormones and growth factors) versus slow, intrinsic signaling, linked to altered metabolism, autophagy, or disease.

## Introduction

In every cell of the human body, intracellular signaling regulates complex functions such as cell growth and differentiation, secretion of hormones or neurotransmitters, and cell adhesion and motility. These responses can be triggered by extracellular stimuli (biochemical, mechanical, and even optical) or result from intrinsic, cell-autonomous signaling. Many signaling proteins are enzymes, capable of efficiently translating intra- or extracellular input into a cellular response. While enzymes such as kinases, phosphatases, and ubiquitin ligases primarily use their enzymatic activity to target and modify another protein (i.e., the substrate), small GTPases, the main focus of this review, are strictly autocatalytic, with their activity resulting in their inactivation, limiting potentially aberrant signaling.

This review focuses on members of the superfamily of small GTPases (153 members) with emphasis on those belonging to the Ras-, Rab-, and Rho-like GTPases [1–3]. These different GTPase families only partially overlap in their control of cellular responses, such as proliferation (mainly Ras-like GTPases), vesicular transport (mainly Rab-like GTPases), and cytoskeletal dynamics (mainly Rho-like GTPases). Given their ubiquitous expression, their key relevance to cells, tissues and organs, and their role in pathologies such as cancer or (chronic) inflammation, tight regulation of small GTPases is an essential aspect of cellular function in health and disease [1].

**Figure 1:**
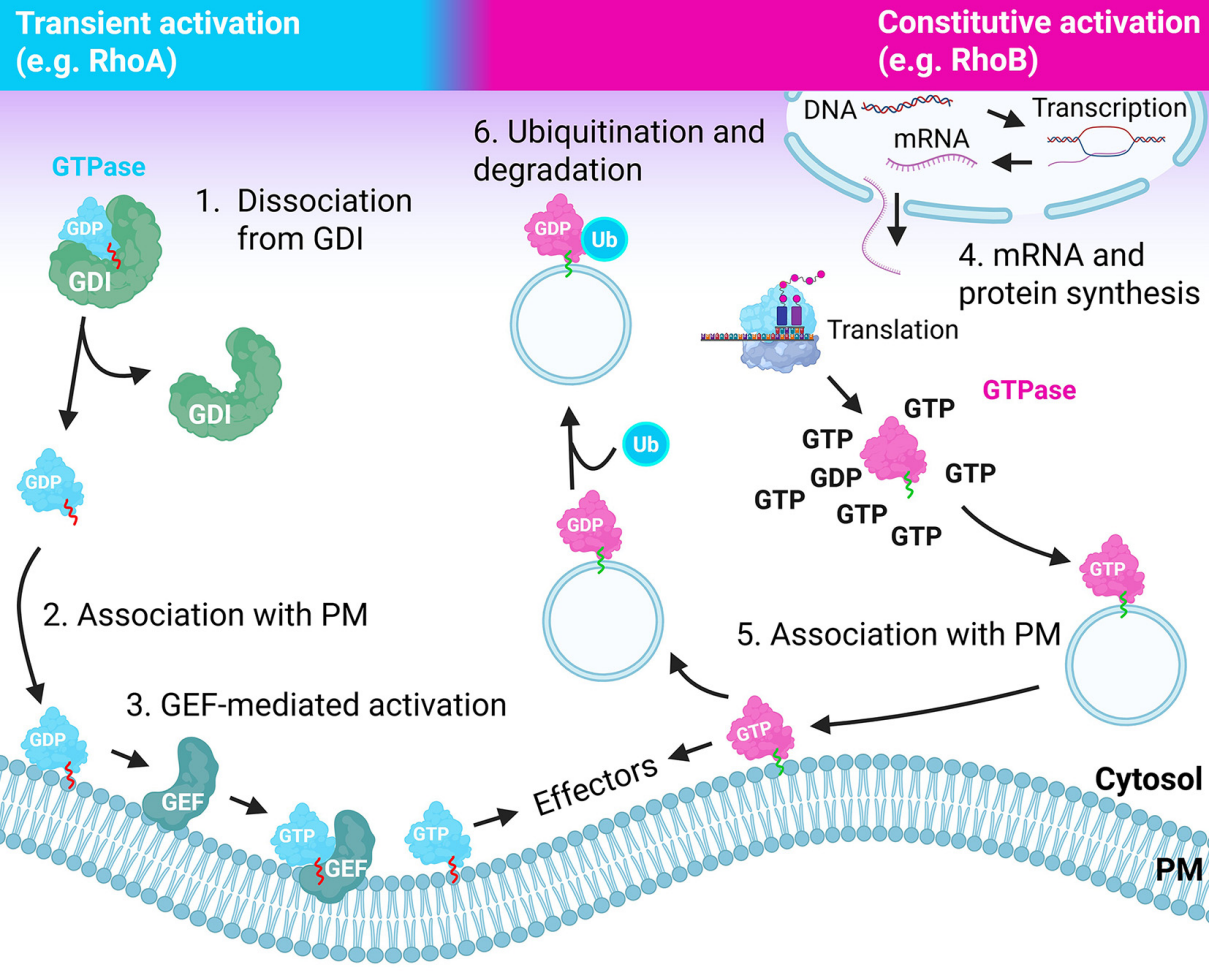
Transient versus constitutive activation of small GTPases The left part of the figure depicts the classical GEF-mediated activation at the plasma membrane, following dissociation from the cytosolic chaperone GDI. The right part of the figure depicts the synthesis of a non-GDI-binding small GTPase, which rapidly becomes lipidated, GTP-loaded, and signaling-competent. Its lipidation targets such GTPase to endosomal membranes and/or the plasma membrane, allowing localized signaling. Subsequent internalization and ubiquitination drive its degradation. Created using Biorender.com.

The canonical mechanism of small GTPase regulation is generally modeled as a switch, with the GTPase alternating between on- or off-states, equivalent to its binding to GTP or GDP, respectively (see below). This switch model has been extensively studied over the past 40+ years and applies to the majority of small GTPases [4]. Consequently, there has been relatively modest attention to alternative means of GTPase regulation, including ubiquitination-mediated or constitutive activation [5] (Figure 1). Following a brief section on the classic switch model of GTPase regulation, this overview focuses on such seemingly uncommon, non-canonical modes of small GTPase activation, which concerns ∼10% of the known small GTPases [5,6]. In this overview, we focus on activation by, in principle, non-pathologic modifications, as mutation-induced GTPase activation has recently been expertly reviewed elsewhere [2,7].

### The canonical mode of small GTPase regulation

Following their synthesis, most small GTPases are modified at their C-terminus by one or more different lipid anchors (e.g., farnesyl, geranylgeranyl, palmitoyl), which allows their targeting to intracellular vesicles or the plasma membrane [8]. This localization is critical for activation, signaling, and inactivation. Generally, small GTPases are considered inactive when bound to GDP. The exchange of GDP for GTP induces a significant conformational change (the ‘on’ state), which allows them to activate specific downstream signaling proteins (effectors) and triggers their intrinsic enzymatic activity, leading to the hydrolysis of the bound GTP to GDP (the ‘off’ state). This cyclic control of small GTPase activation and signaling requires a group of proteins that promote the exchange of bound GDP for GTP. GTP is more abundant in the cytosol as compared with GDP, so opening the nucleotide-binding pocket of the GTPase by such a guanine nucleotide exchange factor (GEF) is sufficient to release the bound GDP and stimulate GTP loading. There is a large number of GEFs for small GTPases (>100, including 85 Rho GEFs), of which most, if not all, reside and function at intracellular vesicles or the plasma membrane, like the GTPases themselves [4,9].

The activated GTPase will interact with membrane-associated effectors in the vicinity, triggering downstream signaling. Small GTPases are poor enzymes that, without help, would remain in the active, GTP-bound state for considerable time [10]. Thus, cells are equipped with another class of membrane-associated, regulatory proteins, the GTPase-activating proteins (GAPs, >150 members [11]). GAPs accelerate the GTPase’s hydrolysis of bound GTP, switching the GTPase off. This ensures a limitation of their output, which is generally accepted to be essential to allow spatially and temporally restricted cellular responses.

The regulation by GEFs and GAPs is known as the *enzymatic cycle* of GTPase (in)activation. In addition, for Rab and Rho small GTPases, there is a parallel cycle, which concerns the localization of the GTPase in the cytosol or the (plasma) membrane. Key to this *spatial cycle* is a small group of cytosolic proteins called GDIs (guanine nucleotide dissociation inhibitors, 3 members), which have a high affinity for GDP-bound GTPases [12,13]. The GDIs secure a large cytosolic pool of inactive GTPases, available to mediate fast responses upon external stimulation, and protect GTPases from degradation. Consequently, GDI-binding GTPases have a relatively long half-life compared with non-GDI-binding GTPases, as discussed below [14,15].

The binding of small GTPases to a GDI involves both protein–protein interactions and docking of the GTPase C-terminal lipid anchor to a binding pocket within the GDI [16]. This explains why a lipidated, GDI-associated GTPase cannot bind to a membrane at the same time. However, the way in which an inactive, GDI-bound, and thus cytosolic GTPase switches to a membrane-bound, active form has been largely unclear and subject to various models. Recently, Armstrong et al. [17] showed, focusing on Cdc42 and using *X. laevis* oocytes as well as reconstituted membranes, that GTPase dissociation from the GDI is a spontaneous event that occurs in solution and is accompanied by (i) the association of the GTPase with the plasma membrane followed by (ii) local GEF-mediated activation. This allows a more or less constant supply of inactive GTPases to membranes where the GEFs reside. Interestingly, these authors showed that GEFs do not compete with the GDI for GTPase binding nor actively recruit the GTPase, which was long proposed to drive the spatial cycle. Notably, aside from the cytosolic association to Rho GDI, this mode of regulation following membrane insertion is similar to the cyclic regulation of most Ras GTPases, which lack a specific GDI and are constitutively membrane-bound [18,19].

The canonical mode of regulation, depicting small GTPases as reusable switches, has been well established over the past decades. However, there are also small (atypical) GTPases such as Rnd1-3, RhoB, and RhoH that do not, or weakly, bind a GDI and appear to escape GAP-mediated inactivation. Atypical GTPases can also be differentiated based on their lack of intrinsic GTP hydrolysis capacity or an increased intrinsic nucleotide exchange rate [20]. These features make them behave as constitutively active signaling proteins. Such GTPases require other modes of regulation, such as proteasomal degradation or phosphorylation [21–26]. There are also several post-translational modifications that trigger GEF-independent GTPase activation and increased, sustained signaling. This may result from increased nucleotide exchange and thus increased GTP-loading or reduced sensitivity to GAP-stimulated GTP hydrolysis [5,27] (Figure 1).

The remainder of this review focuses on such alternative, non-canonical modes of GTPase activation. We aim to underscore that, while the classic switch model may apply to many well-studied small GTPases, regulation by alternative means, albeit less extensively described and seemingly anecdotal, may be more abundant than generally assumed. In addition, we discuss the potential relevance of such alternative GTPase regulation for cellular signaling in health and disease.

### Ubiquitination and activation of small GTPases

**Figure 2:**
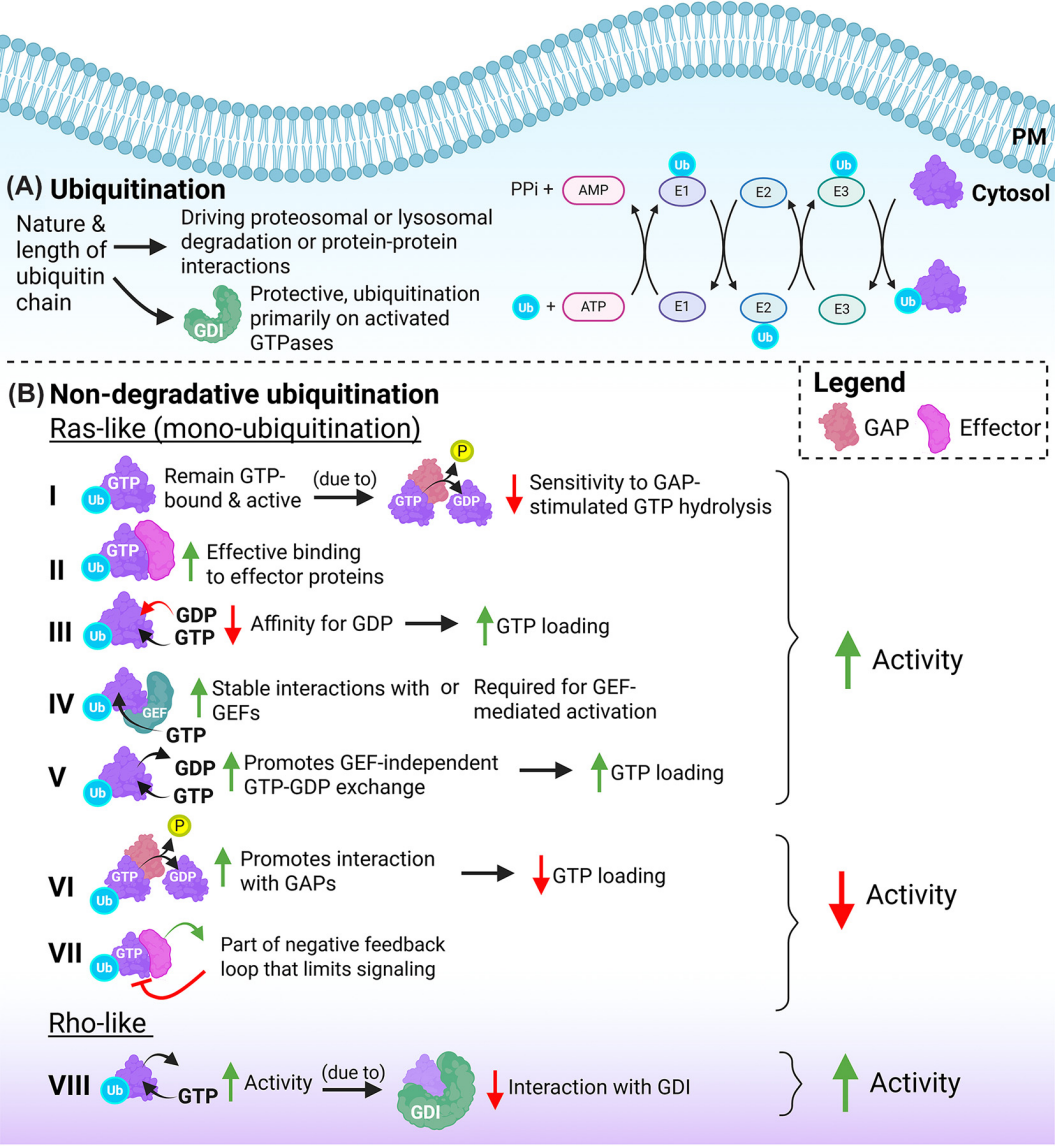
Ubiquitination of small GTPases (**A**) Ubiquitin is transferred from E1- to E2- to E3-ligases and subsequently to the GTPase substrate, unless the GTPase is protected by GDI binding. (**B**) Altered activation by non-degradative ubiquitination in Ras-like and Rho-like GTPases. Mono-ubiquitinated Ras-like GTPases remain (I) GTP-bound and active due to reduced sensitivity to GAP-stimulated GTP hydrolysis, (II) bind more effectively to effector proteins, and (III) have a decreased affinity for GDP, resulting in increased GTP loading. Mono-ubiquitination of Ras-like GTPases also (IV) stabilizes interactions with GEFs and (V) promotes GEF-independent GTP–GDP exchange, leading to increased GTPase signaling. Conversely, mono-ubiquitination of Ras-like GTPases (VI) promotes interactions with GAPs and is part of (VII) a negative feedback loop that limits signaling, reducing GTPase activity. Non-degradative ubiquitination of Rho-like GTPases (VIII) has been associated with increased activity due to reduced interaction with RhoGDI. Created using Biorender.com.

The covalent modification of protein substrates by the 76 amino acid peptide ubiquitin (ubi) is a very common event, driven by the family of ubiquitin ligases (∼600 members). Once a first ubiquitin (ubi) is linked to a substrate, usually via a solvent-accessible Lys (lysine) residue, this ubiquitin can be ubiquitinated as well, driving ubi-chain formation and elongation [28–30]. Ubiquitination serves different purposes, depending on the site at which the substrate is modified as well as the nature and length of the ubiquitin chain, and may either drive proteasomal or lysosomal degradation or protein–protein interactions [31]. Ubiquitination leading to degradation or altered function (through non-degradative ubiquitination) is most extensively studied for members of the Ras family, albeit that such modification of Rho-like GTPases is now also well-established [23,32,33] (Figure 2).

### Ras GTPase activation and ubiquitin

Sasaki et al. [34] showed that K-Ras and H-Ras are ubiquitinated at K104 and at K147, the latter being a common ubiquitination site also in other small GTPases, as in activated Rac1 [35]. The ubiquitination of Ras at K147 comprises mainly mono-ubiquitin, and stimulates mono-ubi-Ras to remain GTP-bound and active. In line with this, ubiquitinated Ras binds more effectively to effector proteins, such as Raf1 or the p85 subunit of PI3K, which further contributes to its tumorigenic capacity. Mechanistically, the ubiquitination of Ras at K147 was suggested to decrease its affinity for GDP, resulting in increased GTP loading [34]. A subsequent study by Baker et al. [36] used a chemical modification approach to directly link mono-ubiquitin to K147 in K-Ras. Their analysis showed that this K147 mono-ubiquitination increases GTP loading and activation because of reduced sensitivity to GAP-stimulated GTP hydrolysis, mimicking the consequences of Ras oncogenic mutations [2]. A more recent study analyzed the effects of mono-ubiquitination of K-Ras at K104, a site also targeted for PTMs such as acetylation, and found that this modification does not so much alter Ras function, but rather stabilizes interactions with GEFs, such as SOS [37,38]. Regulation by mono-ubiquitination is partly GTPase-specific, as mono-ubiquitination of H-Ras at K117 promotes GEF-independent GTP–GDP exchange and thus stimulates activity in a different way than K147 mono-ubiquitination [3,36,39]. It is relevant to note that the K117 residue is well conserved, suggesting its mono-ubiquitination may be a more common way to activate GTPases of different families [3].

Non-degradative ubiquitination appears to be a more common event regulating several types of small GTPase. Sewduth et al. [40] analyzed the ubiquitome of human endothelial cells (ECs), stimulated with the PIEZO-1 activator Yoda1, mimicking the mechano-stimulation by blood flow. These authors found that Yoda1 increased ubiquitination of GTPases of the Rho, Rnd, Arf, Rap, and Rab families, while their protein expression levels (i.e., their degradation) remained unaltered. Focusing on the Rap1 ubiquitination at K31, which lies within the Rap1 effector domain, these authors showed that this did not impair Rap1 activation, but increased its interaction with the effector protein RASIP, but not its effector Talin-1. This suggests that non-degradative Rap1 ubiquitination serves to fine-tune signaling output, similar to what was described for Rab5, where mono-ubiquitination at K140 or K165 controls either altered effector interaction or nucleotide exchange, respectively [40,41].

Conversely, mono-ubiquitination of N-Ras at K128 does not interfere with effector (i.e., Raf1) interactions, but increases its GTPase hydrolysis activity through increased interaction with RasGAP proteins (NF1 and RASA1^GAP^) [42]. Additional studies showed that K128 ubiquitination is part of a negative feedback loop that limits N-Ras-mediated signaling, for example, towards ERK1/2. In addition, expression of a K-RAS-G12D/K128R mutant in pancreatic cancer cells showed that loss of ubiquitination, and thus of GAP-mediated inactivation, stimulated the growth of cancer cell colonies and xenografts. This indicates that K128 mono-ubiquitination restricts Ras signaling through non-degradative ubiquitination, even in the context of a constitutively activating mutation.

The Ras-related GTPase Rap2a is subject to non-degradative ubiquitination by a Cullin5-based E3 ubiquitin ligase complex, which also comprises the small GTPase Rab40b [43]. The ubiquitination of Rap2a, which occurs at K117, K148, and K150, is required for Rap2a recruitment to the plasma membrane and for its recycling between the plasma membrane and early endosomes. This plasma membrane targeting is unusual, as for many membrane-associated proteins, ubiquitination triggers internalization. In addition, these authors showed that Rap2a ubiquitination is required for its activation and signaling towards directional cell migration. In a follow-up study, the same authors showed that Rab40b-Cul5-mediated Rap2a targeting to the plasma membrane supports migration by inhibiting RhoA (via ArhGAP29) in lamellipodia and that ubiquitination of Rap2a is conditional for its GEF-mediated activation. Strikingly, a ubiquitin-deficient Rap2a mutant, which also carried a GAP-insensitive Q63E mutation, was found to be inactive rather than constitutively active. This data supports the notion that non-degradative ubiquitination of Rap2a is required for its GEF-dependent GTP loading, activation, and consequently prolonged resident time at the plasma membrane [43,44]. This mechanism appears to be fundamentally different from, for example, the K147 ubiquitination in K-Ras, which drives increased activation and signaling by rendering the GTPase GAP-insensitive.

### Rho GTPase activation and ubiquitin

The ubiquitination of various RhoGTPases has been well established over the past 15–20 years [21,23,45]. Many of these ubiquitination events cause proteasomal or lysosomal degradation and occur primarily on activated GTPases. This explains, for those GTPases that bind to a GDI, the protective role of GDI association that was previously described [14,15]. However, several studies identified non-degradative ubiquitination of Rho GTPases, including such modification on Rac1, RhoA, and Cdc42 in activated CD4+ T cells [46,47]. Dybas et al. showed that, while basal ubiquitination of cellular proteins already concerned ∼50% non-degradative modifications at baseline, this fraction increased to ∼65% after T-cell receptor stimulation. This data underscores, also for Rho GTPases, the abundance of protein ubiquitination, which is not linked to proteasomal or lysosomal degradation [46].

The Rho GTPase RhoC and, to a limited extent Cdc42, are targeted by the ubiquitin ligase LNX1, which stimulates proliferation of neural stem cells [47]. The primary target residue in RhoC for this non-degradative ubiquitination was identified as K188. This residue is a serine residue in RhoA and is known to be targeted by regulatory phosphorylation by protein kinase A, promoting RhoA stability [48,49]. Interestingly, RhoC K188 ubiquitination increased the level of active RhoC, which was likely due to a reduced interaction of RhoC with RhoGDI. The biological consequence of this regulatory event was an increase in actin stress fiber formation in neural progenitor cells [47].

In a similar study focused on Rac1, Li et al. [50] argued that the ubiquitin ligase TRAF6 mediates Rac1 ubiquitination and activation by targeting K16. However, this conclusion was based on expression of a Rac1K16R mutant, which is deficient in GTP-loading. Since Rac1 activation is required for its ubiquitination [35,51], mutants that block GTP loading and activation will also not be ubiquitinated. Thus, whether K16 in Rac1 is a site for regulatory ubiquitination remains unclear. Finally, it is relevant to mention that the atypical RhoBTB GTPases can, analogous to Rab40b, be part of Cullin3 ubiquitin ligase complexes [52,53].

## Lactylation

**Figure 3:**
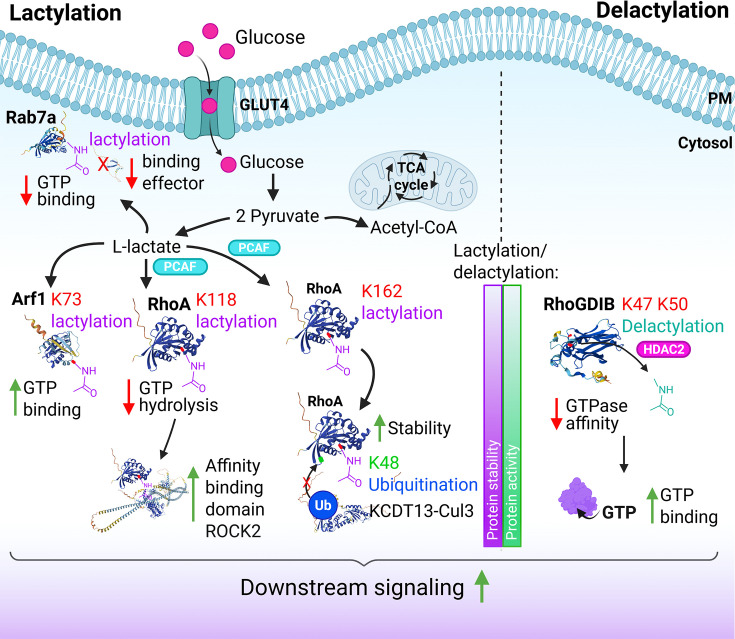
Lactylation of small GTPases Overview of known lactylation sites in Rab7a, Arf1, and RhoA. Lactylation and delactylation can promote activity and stability, among others, by interfering with ubiquitination. Moreover, increased interactions with effector proteins such as ROCK2 increase downstream signaling. The deacylation of RhoGDIB (right part of figure) is proposed to reduce its affinity for bound GTPases, increasing their GTP-binding. Protein structures were adapted from the AlphaFold Protein Structure Database accessed on 6 March 2026: Arf1 (AF-P84077-F1-v6), Cul3 (AF-Q13618-2-F1-v6), KCTD13 (AF-Q8WZ19-F1-v6), Rab7a (AF-P51149-F1-v6), RhoA (AF-P61586-F1-v6), RhoGDI2 (AF-P52566-F1-v6), RILP (AF-Q96NA2-2-F1-v6), and ROCK2 (AF-O75116-F1-v6). Created using Biorender.com.

Another intriguing mode of small GTPase activation, as recently identified for RhoA by Ma et al., is lactylation [54] (Figure 3). Lactylation is increasingly recognized as a relevant post-translational modification that contributes to cell signaling and, for example, tumorigenesis [55]. Lactylation is linked to the Warburg effect and results from the conversion of pyruvate, generated by glycolysis, to lactate, a metabolic pathway that is up-regulated in, among others, tumor cells [56]. Endothelial cells are largely glycolytic [57], which makes it reasonable to assume that lactylation may be particularly relevant for this cell type. Importantly, lactate can also be released into the circulation, for example, in inflammatory conditions such as sepsis, and thus reach the endothelium via this route. This may be particularly relevant for endothelial integrity, as extracellular lactate was recently shown to increase vascular permeability via a G-protein coupled lactate receptor (GPR81)-cAMP-ERK-calpain-VE-cadherin pathway. This pathway drives cleavage and endocytosis of VE-cadherin, the main endothelial cell-cell adhesion protein, with a consequent reduction of endothelial cell–cell contact [58,59].

Regarding its regulation of RhoA, Ma et al. showed that lactylation occurs at K118 and K162, which results in constitutive activation of RhoA and increased signaling to Myosin Light Chain 2 (MLC2) [54]. Lactylation of K118 was found to increase the interaction between RhoA and its effector ROCK2 as a result of strongly reduced GTP hydrolysis. Strikingly, 3D modeling showed that lactylated K118 in RhoA was structurally very similar to known oncogenic and activating mutations in RhoA at that position (K118N, K118R). The lactylation of K162 in RhoA was found to interfere with its K48-ubiquitination by the KCTD13-Cul3 ubiquitin ligase and the consequent proteasomal degradation. K162 lactylation thus results in increased RhoA stability. Since lactylated RhoA was found to be more stable and more active, and RhoA lactylation is increased in breast cancer, this modification was suggested to represent a metabolically driven alternative for oncogenic mutations [2,54].

Most Rho-like small GTPases carry a lysine at the corresponding position of K118 in RhoA, B, and C, except for the Cdc42-like subgroup. RhoA, B, and C also encode a lysine at position 162. This indicates that in cells with increased lactate production, several of these GTPases may become lactylated simultaneously, suggesting that increased tumor cell metastasis may also involve lactylation of migration-regulating GTPases, such as RhoC [60].

In line with this notion, RhoA is not the only small GTPase for which lactylation is described. A recent study focusing on mitochondrial transfer between astrocytes and neurons showed that the LDL-receptor-related protein LRP1 inhibits lactylation of the small GTPase Arf1, a key regulator of vesicular transport [61]. In cells lacking LRP, Arf1 lactylation at K73 was increased, as was Arf1 GTP binding. This indicates that, like for RhoA, Arf1 lactylation promotes its activity and downstream signaling. The biological relevance of this finding lies in the observation that an associated reduction of mitochondrial transfer from astrocytes to damaged neurons was found to exacerbate ischemia-reperfusion injury in the brains of mice [61].

The link between lactylation and increased activation appears not to be true for all GTPases. In hepatocellular carcinoma cells, release of tumor-derived exosomes is under the control of Rab27A and Rab7A, as these control transport of multivesicular bodies to and from the plasma membrane. Lactate was found to reduce the level of GTP binding to Rab7A, but not Rab27A [62]. Similarly, lactate reduced the interaction between Rab7A and its effector Rab-interacting lysosomal protein (RILP). Several lysine residues at positions 21, 31, and 32 were implicated in the effects of lactylation on Rab7A GTP binding and activity. Thus, while lactylation of RhoA and Arf1 was found to stimulate their activity, lactylation of Rab7A reduced its activity [62].

Finally, a very recent, related study implicates lactylation of the tumor suppressor RhoGDIB (also known as RhoGDI2) in the context of bladder cancer metastasis [63]. Here, removal of lactate (delactylation) by HDAC2 from lysines 47 and 50 in RhoGDIB reduces its affinity for several small GTPases, including Rac1 (Figure 3). The result is that Rac1 GTP-loading increases, which is linked to increased cisplatin resistance in bladder cancer. The study also identified interactions of RhoGDIB, which binds Rac1 and Rac3 [12], with RhoC and Cdc42, so it cannot be excluded that delactylation of RhoGDIB would also activate additional GTPases that are functionally linked to tumor cell metastasis.

## Redox regulation

**Figure 4:**
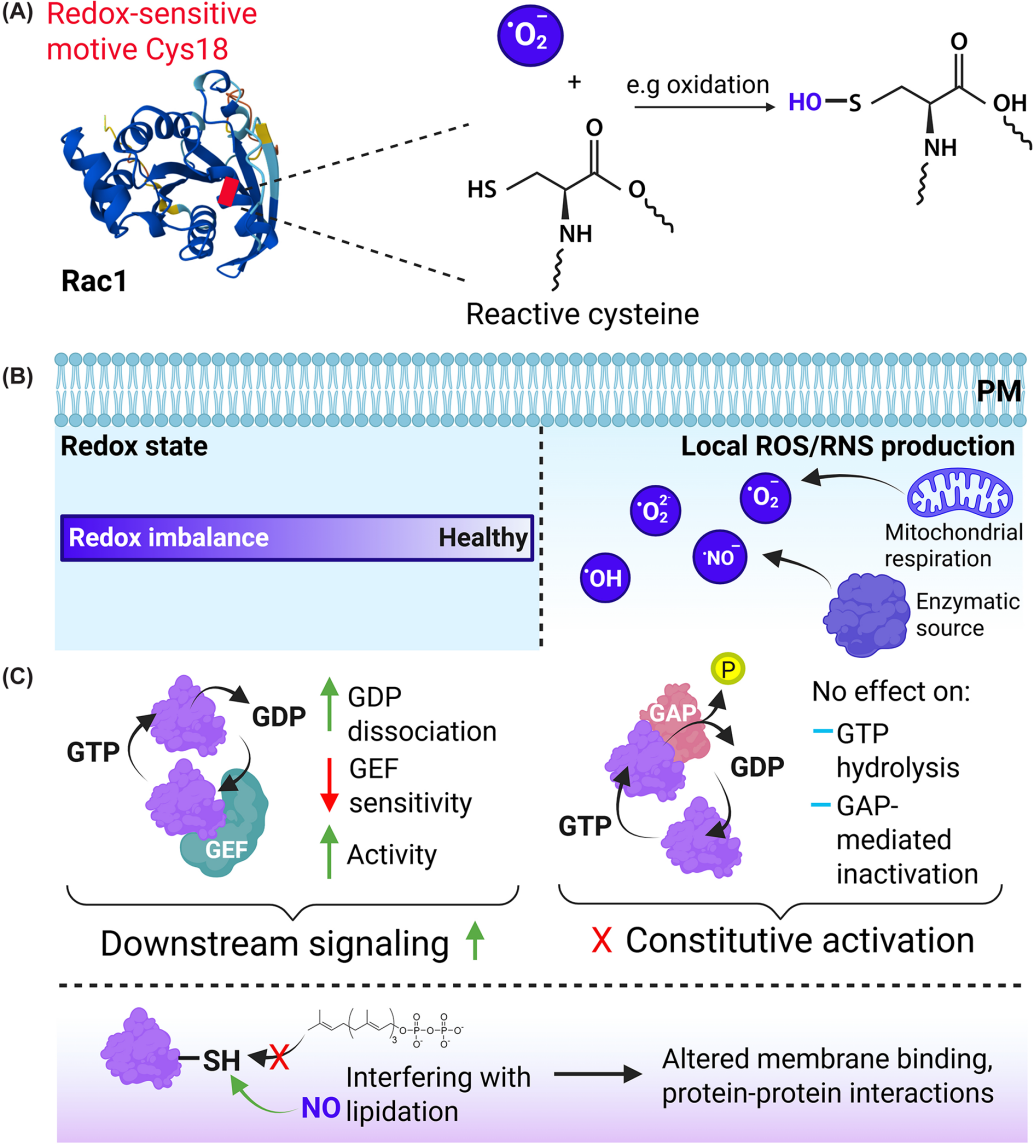
Redox regulation of small GTPases (**A**) Example of oxidation of the redox-sensitive motif in Rac1. (**B**) Redox regulation is controlled by the redox state of the cell, reflecting a healthy or diseased (imbalanced) state, or by local production of ROS or RNS. (**C**) The different effects of oxidation-mediated post-translational modification of small GTPases and the effects on their activity and downstream signaling. Note that site-specific oxidation may interfere with normal lipidation, altering GTPase targeting and signaling capacity. Rac1 structure was adapted from the AlphaFold Protein Structure Database accessed on 6 March 2026 (AF-A4D2P0-F1-v6). Created using Biorender.com.

It is known already for >20 years that regulating small GTPase activation and output can occur via redox regulation [64,65]. Such oxidation-based means of control (including S-nitrosylation) were originally described for Ras GTPases and subsequently also for members of the Rho family [66,67]. In fact, Heo and Campbell already in 2005 proposed that redox agents represent an alternative mechanism to GEFs by stimulating guanine nucleotide dissociation and activation of Rho GTPases [67]. This alternative mode of regulation is not so much controlled by outside stimulation, but rather by changes in the redox state of the cell, for instance during hypoxia, and/or the local production of reactive oxygen species (ROS) or reactive nitrogen species (RNS) (Figure 4).

The S-nitrosylation of Ras GTPases has been well established and has been recently reviewed [68]. Briefly, Ras S-nitrosylation mainly occurs at Cys118 and increases nucleotide exchange, leading to activation and increased signaling. In addition, this modification alters subcellular targeting of Ras. Different from Cys118 targeting in Ras, Rho GTPases contain a redox-sensitive motif at amino acids 10–17, at the end of the so-called P-loop, followed by the reactive cysteine at position 18 (Rac1 numbering). Rac1 is modified by oxidation through the cellular oxidant glutathione directly, as shown by Hobbs et al. using various Rac1C18 mutants [69]. The present study found that in human articular chondrocytes, glutathione-mediated oxidation of Cys18 in Rac1 (or a Rac1C18D mimic in HEK293 cells) accelerated GDP dissociation ∼200-fold with concomitantly increased GTP-loading and activity, and insensitivity to additional GEF-mediated exchange. Intriguingly, while nucleotide exchange was much accelerated, the GTP hydrolysis and GAP-mediated inactivation remained similar, showing that oxidant-modified, active GTPases remain sensitive to inactivation. This also means that while oxidation may increase activation and downstream signaling, it does not lead to constitutive activity [69].

For RhoA/B/C, the situation is less straightforward, as they firstly encode a Cys at position 20, homologous to Cys18 in Rac1/Cdc42; their oxidation could secondly induce disulfide bonds with a cysteine at position 16, leading to interference with nucleotide binding and inactivation [64,65]. However, modification of Cys20 in RhoA by itself is activating, so the effect of oxidant-mediated modification of RhoA/B/C GTPases is complex [70]. Interestingly, Cys16 without an accompanying Cys20 is conserved in RhoD, RhoE/Rnd3, and RhoF, suggesting that there may be a role for Cys16 oxidation that has not yet been elucidated [64].

A recent study identified regulation of the Rab5 GTPase by reversible S-nitrosylation, induced by NO, in phagocytes [71]. This modification on Cys212 and Cys213, within the Rab5 C-terminal region, promotes Rab5 activation and stimulates Rab5-mediated phagocytosis, as shown in ectopic expression studies and in mice. Conversely, an activated mutant of Rab5 (Q79L) binds more effectively to iNOS (induced nitric oxide synthase) and also is more nitrosylated, suggesting a positive feedback loop in this mode of regulation of Rab5. The nitrosylation of Cys212/213 would interfere with, or be an alternative for, their geranylgeranylation. The authors suggest that specific conditions, related to cellular levels of cholesterol or its biosynthesis, e.g., the use of statins, may cause more non-lipidated Rab5 to be available for nitrosylation. Membrane binding of such alternatively modified GTPase would be more dependent on protein–protein interactions rather than on lipid-mediated targeting [71] (Figure 4).

## Palmitoylation and constitutive activation

**Figure 5:**
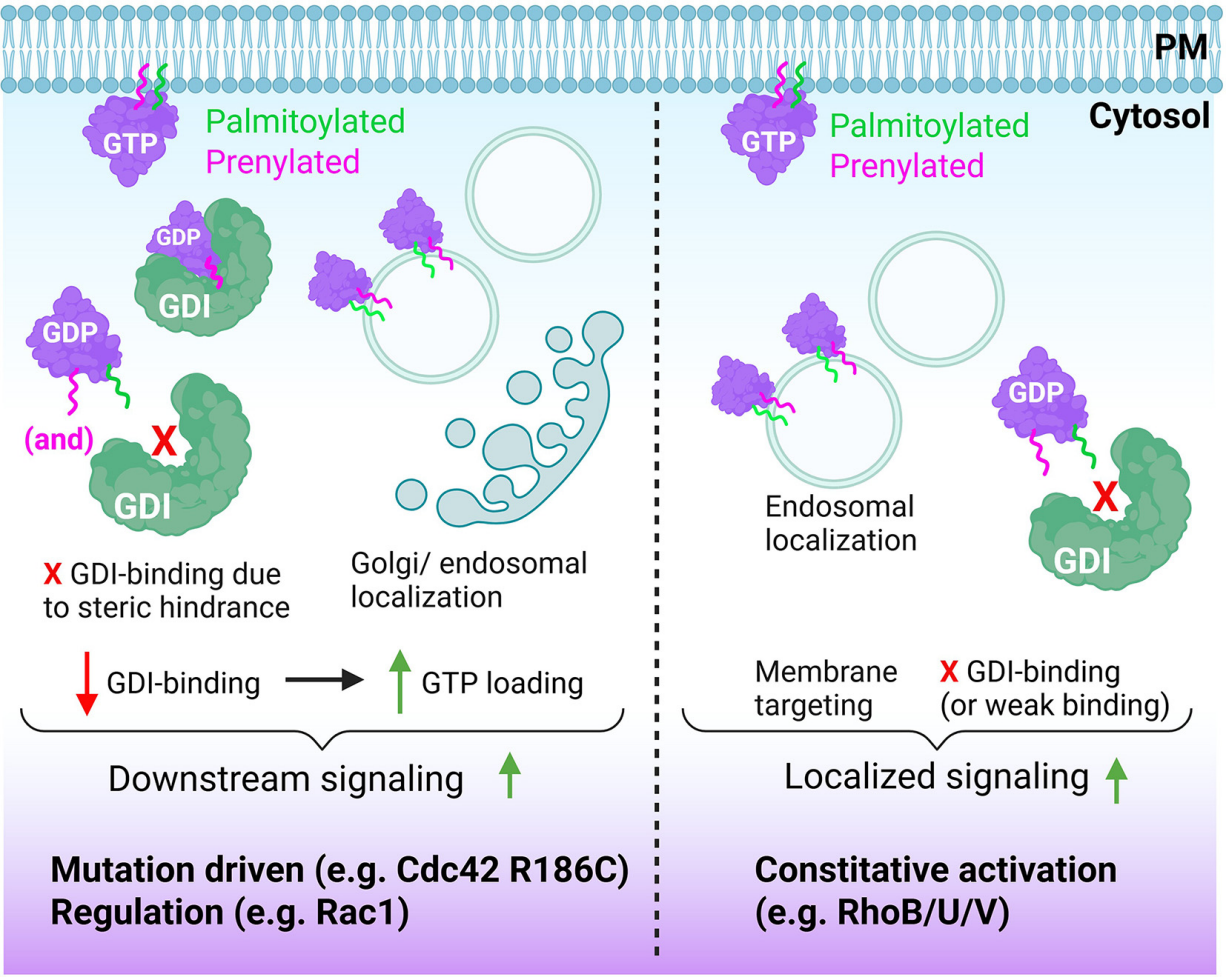
Palmitoylation of small GTPases drives (localized) signaling Left, A non-palmitoylated, prenylated GTPase will associate with the GDI. Conversely, palmitoylation due to a mutation or regulatory mechanisms will interfere with GDI binding and drive the GTPase directly to internal membranes. Lack of GDI binding is associated with increased GTP loading and signaling. Right, some GTPases are constitutively palmitoylated, resulting in lack of GDI binding and increased, constitutive signaling. Note that for RhoB, a weak interaction with RhoGDI-3 has been detected, suggesting that part of its regulation may be GDI-based. Created using Biorender.com.

The intracellular targeting of small GTPases requires the combined presence of a polybasic region, mostly in the C-terminus, together with specific C-terminal lipidation, such as farnesylation or geranylgeranylation [2,72]. In addition, a growing body of evidence shows that also the reversible palmitoylation of a significant number of small GTPases contributes in several ways to their targeting, activation, and signaling (Figure 5).

### Cdc42

Cdc42 is an interesting example of differential control by palmitoylation, as there exist two isoforms that arise from alternative splicing. These isoforms are expressed either ubiquitously or selectively in the brain. The main, ubiquitous Cdc42 isoform is geranylgeranylated at C188, while the brain isoform (bCdc42), which encodes an alternative CCaX box, is also constitutively palmitoylated at the adjacent C189. Moreover, its C-terminus is not processed by the usual removal of the AAX portion and carboxymethylation of the C-terminal cysteine [73]. These authors also showed that palmitoylation in fact requires prenylation, and that palmitoylated bCdc42 does not bind to RhoGDI and is stably associated with membranes, in line with the steric hindrance of GDI binding by the palmitoyl. Moreover, interfering with its palmitoylation reduced the signaling capacity of the bCdc42 isoform, possibly as such a non-palmitoylated form can bind to RhoGDI, reducing its availability for activation. Similar to the brain isoform of Cdc42, palmitoylation is constitutive for several other small GTPases, including H-Ras, N-Ras, K-Ras4A, RalA, and RalB, as well as RhoB, TC10, RhoU, and RhoV [2,74]. Interestingly, palmitoylation of the ubiquitous Cdc42 isoform was reported in a patient with a Cdc42 R186C mutation. The mutated Cdc42 showed a loss of GDI-binding, was constitutively localized to the Golgi, induced hyperactivation of NFkappaB, and caused a severe skin rash as well as systemic inflammation. This suggests increased and mislocalized signaling by the mutated, palmitoylated Cdc42 [75].

### RhoU/RhoV

The Cdc42-related small GTPase RhoU (also known as Wrch1 [76]) belongs to the group of ‘atypical’ GTPases. Several recent studies have shed new light on the mechanisms that regulate RhoU and its important role in, among others, planar cell polarity in epithelial cells [77]. Like RhoV (Wrch2), RhoU shows a fast guanine nucleotide exchange rate due to a tyrosine instead of a phenylalanine at the position equivalent to F28 in Rac1 or Cdc42, which also show a fast-cycling phenotype, similar to the oncogenic Rac1P29S mutant [78,79]. Similarly, since both RhoU and RhoV are palmitoylated, these GTPases do not bind Rho GDI (with the exception of RhoV, but not RhoU, binding to RhoGDI3 [12]). Thus, their palmitoylation allows endosomal localization and rapid, constitutive activation, which explains their non-canonical (atypical) behavior and regulation [80].

### Rac1

Navarro-Lérida showed in 2012 that Rac1, which is modified by geranylgeranylation at Cys189, can additionally be palmitoylated at its C-terminus (Cys178) [81]. Ectopic expression of Rac1 in Cos7 cells showed that inhibition of palmitoylation reduced both Rac1 association with liquid-ordered membrane domains and its GTP-loading. Moreover, inhibition of Rac1 palmitoylation, using a Rac1 C178S mutant, did not restore the capacity for spreading and migration as induced by wtRac1 in mouse embryonic fibroblasts. Thus, palmitoylation significantly and positively contributes to Rac1 activity and signaling.

The levels of Rac1 palmitoylation in these experiments were found to be relatively low, despite its relevance for Rac1 biological activity. This suggests that the subfraction of endogenous Rac1 that is palmitoylated induces most of the signaling, or that Rac1 palmitoylation occurs transiently and may be detectable only as a fraction of the total pool in biochemical analyses. In line with the latter, the authors conclude that transient palmitoylation of Rac1 serves to effectively and dynamically alter membrane organization and cell signaling, which would not be as effective if the protein were constitutively associated with these ordered microdomains [81].

While the palmitoylation of Rac1 has received little attention since the studies by the Del Pozo group, its role in cardiomyocytes was recently analyzed in more detail. This was based initially on findings regarding the S-acyltransferase zDHHC3, the ectopic expression of which mediates dilated cardiomyopathy [82]. zDHHC3 induces S-palmitoylation of, among others, Rac1 and promotes its membrane association, GTP-loading, and activity towards effectors such as Pak1. Intriguingly, overexpression of active Rac1 was previously shown to drive dilated cardiomyopathy [83]. A follow-up study showed that palmitoylation of Rac1 in cardiomyocytes plays in fact a more protective role, countering hyperphosphorylation by PKA, in conditions of cardiac stress [84]. Using Rac1C178S conditional knock-in mice, these authors showed that, while the palmitoylation-resistant Rac1 protein was more abundant in the cytosol, likely due to GDI binding, there was no obvious phenotype in the absence of a stimulus in either whole-body- or cardiomyocyte-specific knock-in animals. In contrast, angiotensin II infusion induced a significantly stronger cardiac hypertrophy in the knock-in animals, indicative of a more protective role of Rac1 palmitoylation in the pressure-overloaded heart [84].

### RhoB

Although RhoB is highly homologous to the closely related GTPases RhoA and RhoC, it clearly stands out as it is, in contrast with RhoA and RhoC, palmitoylated on Cys189 and Cys192, in addition to its isoprenylation (either geranylgeranyl or farnesyl) on Cys193 [85]. The palmitoylation of RhoB prevents its binding to RhoGDI, although a weak interaction with the endosome-associated RhoGDI-3 expressed mainly in brain and pancreas, but not to RhoGDI-1 or -2, has been described following ectopic co-expression in HEK293T cells [12]. Consequently, RhoB, which localizes to both endosomes and the plasma membrane, has all the hallmarks of a constitutively active GTPase, although regulation by the GEFs XPLN (also known as ARHGEF3) and Solo as well as the GAPs p190BRhoGAP and DLC3 has been described [86–89]. However, RhoB is well known for its short half-life (1–2 h) as compared with RhoA (10–12 h), which is well in line with its lack of GDI binding and consequent susceptibility to degradation [90,91]. Indeed, our lab and others showed constitutive RhoB ubiquitination and lysosomal degradation in unstimulated human EC, with the Cullin3 E3 ligase KCTD10 as an important regulator [24,92,93]. We and others have further shown that either inhibition of proteasomal/lysosomal degradation or of E1- or E3-ubiquitin ligases rapidly (within 4–6 h) increases RhoB protein levels [24,93]. Associated with this up-regulation was a strong increase in F-actin stress fiber formation and contractility. While these are classical RhoA-driven responses, RhoB has the exact same effector region as RhoA and is likely to activate a similar set of effectors, including ROCK and MLC2 [85]. Importantly, we also showed that in EC the increase in RhoB protein expression following the inhibition of ubiquitination is paralleled by an equivalent increase in active, GTP-bound RhoB [90]. Apparently, there is no, or insufficient, GAP activity available to down-regulate this pool of activated RhoB. Clearly, the palmitoylation of RhoB can explain this behavior and mode of regulation, suggesting RhoB is not only an atypical, but also a constitutively active GTPase (Figure 5).

### Constitutive activation

Similar to RhoB, RhoU, and RhoV, RhoH and the Rnd GTPases are also considered atypical. These GTPases are not subject to canonical cycling due to the fact that they do not encode a glycine residue at the G12-equivalent position [94–96]. Mutations at position G12 (or equivalent) are well known to be oncogenic in Ras GTPases and lead to constitutive activity in RhoA and Rac1, because the mutation impairs GTP hydrolysis [2]. Similar to other non-GDI binding GTPases, the Rnds are GTP-loaded upon synthesis and effectively act as constitutively active GTPases with a short half-life of ∼2.5 h in keratinocytes and HeLa cells. Interestingly, membrane association of Rnd3 was required for its degradation, as a Rnd3(C241S) cytosolic mutant was significantly up-regulated, albeit that the relevant mechanism or ubiquitin ligase was not identified [94]. Also, ROCK activity was found to stimulate Rnd3 turnover via a potential feedback loop driven by p190RhoGAP-RhoA signaling, which is induced by Rnd3 activity. This suggests that Rnd3 phosphorylation by ROCK may be instrumental in its turnover, which is in line with data from Riento et al., who identified Rnd3 as a substrate for ROCK phosphorylation on Ser-11, which drives Rnd3 to the cytosol and stabilizes its expression [97]. Constitutive activation has also been established for RhoD and its close relative RhoF, which do express a Gly at the G12-homologous position (Rac1 numbering). However, these GTPases show high intrinsic exchange rate, which keeps them largely in the GTP-bound form [98,99].

RhoH is a (largely) hematopoietic cell-specific GTPase, which is GTPase-deficient and thus constitutively active, due to changes in the sequence of the G3/Switch II motif and the absence of the critical Gly12 residue. Due to its constitutive activity, RhoH is also insensitive to GAP-mediated inactivation [6], and its output is largely controlled by its expression level. In addition, similar to other constitutively active GTPases, the half-life of RhoH is short (<3 h [100]). In line with this, Troeger et al. [25] showed earlier that a short (6 amino acids) C-terminal insertion in RhoH mediates its lysosomal degradation in T lymphocytes in a pathway related to chaperone-mediated autophagy. Intriguingly, RhoB, but not RhoA or RhoC, also has a short (3 amino acids) insertion in its C-terminus, which is linked to its lysosomal degradation [91,101,102].

Finally, another recent example of non-canonical activity regulation of a GTPase comes from a study in budding yeast [103]. Here, the small, Ras-like GTPase Tem1 signals spindle orientation to the mitotic exit network as part of a pathway regulating genome partitioning between dividing cells. Although Tem1 can undergo GDP/GTP cycling, it does not appear to require a GEF to become activated. Rather, it shows fast intrinsic exchange, which keeps Tem1 mainly in the GTP-bound form. Intriguingly, its GTP-bound state is concentrated at the spindle pole body, through interactions with a GAP complex and its effector Cdc15, and signaling by Tem1 did not change its nucleotide state. Based on additional mutational analyses and localization experiments, the authors conclude that it is Tem1 recruitment and local concentration, rather than its nucleotide-bound state, that are key aspects that control its capacity to signal [103].

## Discussion and conclusions

Research on GTPase signaling has long focused on spatio-temporal regulation, redundancy, and specificity. The studies established that the large repertoire of GEFs and GAPs allows localized, restricted, and stimulus-specific GTPase cycling [104,105]. Regarding the non-canonical modes of activation discussed above, it is not immediately clear why cells would require GEF-independent GTPase activation or express small GTPases that are always in the active, GTP-bound form. Below we discuss two potential explanations for the existence of non-canonical GTPase regulation. To simplify the discussion, we assume there are two modes of GTPase regulation: (i) GEF- and GAP-mediated control and (ii) GEF-independent activation in conjunction with synthesis and degradation-mediated expression control (Figure 1).

### Differential kinetics

The GEF/GAP cycle is assumed to act relatively fast when activating a GTPase that resides at the membrane in the vicinity of a GEF, either because the GTPase is constitutively membrane-associated, as for Ras GTPases, or associates with membranes upon spontaneous dissociation from a GDI [17]. The canonical on/off cycle is, in many systems, detectable at a time scale of seconds to minutes, using either classical biochemical experiments or biosensor studies. For example, thrombin- or LPA-mediated activation of RhoA, which can be induced through p115RhoGEF or GEFH1, is detected within 0.5–5 min and declines again in 20–30 min in EC [106–109]. Also, detection of local activation of RhoA or Rac1 using biosensors in live cells requires second-to-minute resolution [110,111].

This is in marked contrast with the control and signaling by constitutively active GTPases. Their GTP loading and continuous signaling appear to be mostly limited by degradation. The link between (constitutive) activity and consequent ubiquitination and degradation has been firmly established for Rac1 and RhoB. For Rac1, its ubiquitination was established upon its GTP-loading, be it using activated Rac1 mutants or using bacterial toxins such as CNF1, which ADP-ribosylates and thereby activates endogenous Rac1 [35,51,112]. RhoB is palmitoylated and does not, or only weakly, bind to the protective GDI. As a result, RhoB becomes GTP-loaded upon synthesis and is constitutively ubiquitinated and degraded in lysosomes [24,85,91]. It is likely that this is not much different for similarly modified GTPases such as RhoU/V and the Rnd GTPases, as well as for the palmitoylated forms of Rac1 and Cdc42, and the brain Cdc42 isoform.

Thus, for many GTPases, activation increases sensitivity to ubiquitination. However, the duration of the consequent degradation, balanced by GTPase synthesis, may extend well beyond the second-to-minute time scale. For example, we showed that inhibition of ubiquitin ligases in EC markedly increases expression of RhoB within 4–6 h [24,90]. Similarly, pro-inflammatory TNFα stimulation up-regulates constitutive RhoB synthesis in EC, the detection of which also requires at least 4–6 h [24,113].

Together, these findings indicate that while the canonical cycle controls rapid and transient signaling by readily available GTPases, non-canonical regulation is comparatively slow, may in part depend on increased protein synthesis, and could mediate a much more chronic type of signaling, analogous to a stationary engine running for longer times at higher rpm. Such pronounced kinetic differences between the two cycles may thus well relate to the biological responses and (external or internal) stimuli they are associated with.

## Extrinsic versus intrinsic stimulation

It is well established that GEF/GAP-mediated cycling can be triggered by cellular stimuli such as bioactive peptides and growth factors, acting through cell surface receptors. These receptors are likely to reside in the vicinity of GEFs or associate with them in larger signaling complexes [114,115]. Conversely, GEF-independent activation or constitutive signaling may well be more often associated with cell-autonomous events that require much larger time scales. For example, RhoB has been linked to autophagy in several studies because of its association with the regulator Beclin-1 [116] and its role in bacterial infection-mediated induction of autophagic responses [117]. RalB was also linked to Beclin-1, as its deubiquitination by USP33 at K47, within the effector domain, alters its interactions with selected effector proteins. Consequently, this allows RalB to switch from regulating innate immune responses to the induction of autophagy [118]. The constitutively active RhoD also stimulates autophagy by driving autophagosome formation through its control of ATG9 trafficking from the Golgi. Surprisingly, this function was found to be independent of the RhoD nucleotide-bound status [119].

The modification of RhoA and other GTPases by lactylation is clearly linked to cellular metabolism, in this case glycolysis and the production of pyruvate. Changes in metabolic pathways may well be specifically relevant for small GTPase modification and activation on longer time scales. The same holds for ubiquitination, a process linked to cellular quality control and (also) autophagy. Similarly, redox-dependent GTPase activation through oxidation is a consequence of altered cellular metabolism, associated with enhanced production of RNS/ROS, which occurs in response to chronic (stress) conditions such as hypoxia. Finally, also palmitoylation is clearly linked to changes in metabolism and, for example, dyslipidemia [120]. Consequently, protein S-palmitoylation is associated with cardiovascular disease, even though the modification itself is reversible and can be triggered on short time scales [121]. Based on these correlations, it is attractive to propose that GEF-independent, constitutive activation drives cell-autonomous processes related to more sustained conditions and the associated (changes) in metabolic activity.

To extend this one step further, it is also attractive to link slow and chronic GTPase signaling, for example, due to up-regulation of expression, to the induction and persistence of various types of pathology. The inflammation-associated up-regulation, induced by TNFα, of RhoB in endothelium is an intriguing example. RhoB was found to be up-regulated also in chronic kidney disease and RhoB protein is even detectable in the urine of CKD patients [122–124]. RhoB expression increases in response to ROS-induced signaling and various forms of cellular stress, including DNA damage and ischemia [125–128]. Similarly, ubiquitination and degradation by Cullin3 ubiquitin ligases in smooth muscle cells have been linked to hypertension, albeit that in those studies, RhoA, rather than RhoB, was proposed to be the main Cullin3-regulated (i.e., ubiquitinated) GTPase [129,130]. The expression of the constitutively active RhoH is down-regulated upon terminal differentiation of myeloid cells, a response that is dysregulated in acute myeloid leukemia [131]. RhoH may thus, similar to RhoB or RhoU, act as a tumor suppressor [76,132,133].

In yet another recent example of GEF-independent activation, it was shown that R-Ras2, which has a high intrinsic nucleotide exchange rate, associates directly with immunoreceptor tyrosine-based activation motifs (ITAMs) of antigen receptors [134]. This high-affinity interaction requires the R-Ras2 proline-rich region in its hypervariable C-terminus. Intriguingly, nucleotide exchange due to opening up of the nucleotide binding pocket on R-Ras-2 could be induced *in vitro* by an ITAM-peptide, suggesting the antigen receptors themselves could act as GEFs for R-Ras2, not unlike G-protein-coupled receptors acting as GEFs for heterotrimeric G-proteins [135]. This ITAM-based activation represents a very different mechanism of activation compared with the classical GEF-mediated activation by interactions with the GTPase switch I and II regions [9].

Finally, there are situations where GEF/GAP-mediated cycling appears to drive relatively slow responses. A good example is cell migration, where activity cycles of small GTPases on short time scales, observed using biosensors in live cells, control highly localized, sustained morphological events (membrane protrusion, retraction) at the leading or trailing edge [110,136]. Similarly, cytokinesis is known to be mediated by specific GEFs such as Ect2 and GEFH1, activators of RhoA [137]. Thus, there are various examples of relatively slow or sustained cellular responses that require specific GEFs, which would imply that such responses are indeed governed by some form of cycling. This could also indicate that in such processes, it is the GEF, rather than the cycling GTPase, that is activated for extended periods of time [138,139]. However, the molecular mechanisms driving such extended, GEF-mediated signaling are not well established and warrant further detailed experiments.

## Concluding remarks

In this review, we aimed to highlight the relevance of small GTPase regulation via mechanisms that deviate from the classical GEF/GAP-regulated cycle. GTPase activation, induced by different post-translational modifications that change intrinsic GTP exchange or hydrolysis, significantly expands the options for GTPase regulation. Non-canonical and constitutive activation may represent more a dial or dimmer, rather than a switch [5], which would provide a cell more options to either fine-tune signaling output or generate increased signaling over prolonged periods of time. The relevance of such alternative regulation in (cardiovascular) disease, including kidney disease, hypertension, or inflammatory disorders, warrants more in-depth studies. This understudied mode of small GTP activation represents an interesting avenue for future translational research, complementary to studies on mutation-induced, GTPase-driven pathology, such as cancer.

## Open Access

Open access for this article was enabled by the participation of the University of Amsterdam in an all-inclusive Read & Publish agreement with Portland Press and the Biochemical Society.

## Abbreviations

ADP: Adenosine diphosphate
ArhGAP: Rho GTPase-activating protein
ArhGEF: Rho Guanine nucleotide exchange factor
ATG9: Autophagy related protein 9
cAMP: cyclic Adenosine Monophosphate
DLC3: Deleted in liver cancer 3
EC: Endothelial Cell
Ect2: Epithelial cell transforming 2
ERK1: Extracellular Regulated Kinase 1
GAP: GTPase Activating Protein
GDI: Guanine nucleotide Dissociation Inhibitor
GDP: Guanosine diphosphate
GEF: Guanine nucleotide exchange factor
GPR81: G-protein coupled Receptor 81
GTP: Guanosine-5′-triphosphate
HDAC2: Histone DeACetylase2
HEK293: Human Embryonic Kidney cells 293
iNOS: induced Nitric Oxide Synthase
ITAM: Immunoreceptor Tyrosine-based Activation Motif
KCTD10: Potassium Channel Tetramerization Domain 10
LNX1: Ligand of Numb-protein X 1
MLC: Myosin Light Chain
NF1: Neurofibromatosis type 1,
PI3K: PhosphatidylInositol-3-inase
PKA: Protein Kinase A
RASA1: RAS p21 protein activator 1
RILP: Rab-interacting lysosomal protein
RNS: Reactive Nitrogen Species
ROCK: Rho Kinase
ROS: Reactive Oxygen Species
SOS: Son of Sevenless
TNFalfa: Tumor Necrosis Factor alfa
TRAF6: TNF-receptor Associated Factor 6
zDHHC3: Zinc finger DHHC-type palmitoyltransferase 3
